# Long-level Intramedullary Spinal Cord Tumor: A Case Series

**DOI:** 10.31729/jnma.8924

**Published:** 2025-03-31

**Authors:** Namrata Khadka, Anil Suryabanshi, Apurva Shrestha, Kumar Paudel, Sameer Aryal, Binit Jha, Sushil Mohan Bhattarai, Binod Rajbhandari, Rajendra Shrestha, Rajiv Jha

**Affiliations:** 1 Department of Neurosurgery, National Neurosurgical Referral Center, National Academy of Medical Sciences Bir Hospital, Kathmandu, Nepal; 2 Tribhuvan University, Institute of Medicine, Maharajgunj, Kathmandu, Nepal

**Keywords:** *ependymoma*, *intraoperative neuromonitoring*, *long-level intramedullary*, *spinal cord tumor*

## Abstract

Spinal cord tumors, though uncommon, pose significant challenges due to their potential for neurological disability and mortality. Intramedullary spinal cord tumors, particularly Long-level intramedullary spinal cord tumors, present challenging clinical scenarios. Contrast-enhanced Magnetic resonance imaging remains pivotal for radiological evaluation and surgical planning. Notably, aggressive resection is advocated to enhance prognosis, with meticulous attention to preserving neurological function. Advancements in spinal surgery techniques, coupled with intraoperative monitoring, offer promising avenues for improved patient outcomes. We presented three cases of long-level intramedullary tumors, emphasizing the significance of tailored management and presented details, including clinical presentations, radiological findings, and histopathological results.

## INTRODUCTION

Spinal cord tumors are uncommon pathological conditions. Primary spinal cord tumors account for 2-5% of all primary central nervous systems, with intramedullary spinal cord tumors (IMSCTs) comprising one-third of these cases.^1^ Notably, when IMSCTs extend to involve at least five spinal segments, they are termed Long-level intramedullary spinal cord tumors (LIMSCTs).^1^ Though rare, LIMSCT poses significant neurological disability and mortality. However, aggressive and radical resection has shown positive results in preserving neurological function.^2,3^ So, surgery is the standard treatment in most cases. Excision of spinal cord tumors is a challenging procedure because not only the removal of mass but preservation of neurological function is important. Consequently, treatment decisions must be meticulously planned. In this case series, we present our experience derived from managing patients afflicted with LIMSCTs.

## Case Series:

### Case 1

**Figure 1:**
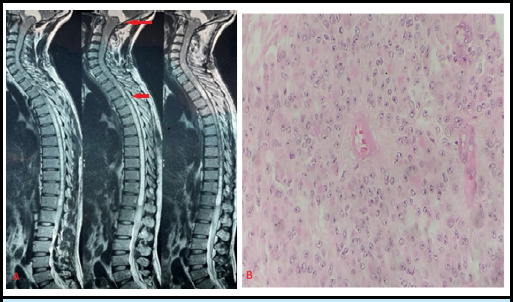
Pane A- MRI T2 sequence showing intramedullary spinal tumor extending from cervico-medullary junction to T4 level. Pane B- Haematoxylin & Eosin stain (magnification x100) showing perivascular pseudo rosettes formation of tumor cells.

A 34-year-old right-handed male from a middle socioeconomic background presented to the Neurosurgical Outpatient department (OPD) with complaints of progressive weakness in both lower limbs for 4 months and the left upper limb for 1 month, with preserved bowel and bladder function. He had no history of fever or trauma. On examination, diminished sensation to pain, temperature, and vibration was noted in the left upper limb. Increased tone was observed in both lower limbs. Power in the left elbow and left wrist was 4/5, with a handgrip strength of 75%. Power in the left hip and left knee was 4/5. Reflexes in the left biceps and left triceps were 3+, while plantar reflexes were bilaterally mute. The rest of his CNS examinations were within the normal range. An MRI of the spine revealed an intramedullary mass extending from the cervico-medullary junction to the T4 level (Figure 1). Surgical excision of the intramedullary mass with laminoplasty was performed, revealing a tumor length of 20.5 cm. The resected mass was sent for biopsy, which reported it to be an Ependymoma WHO grade 2 (Figure 1). The postoperative McCormick grade was II.

### Case 2

**Figure 2:**
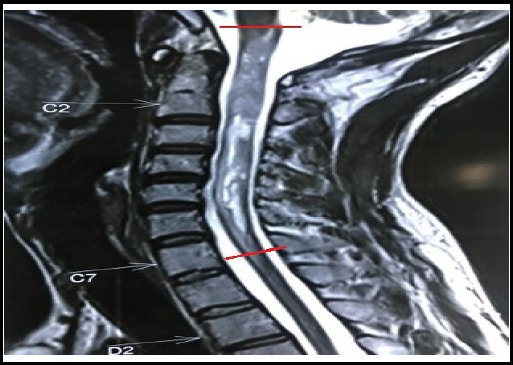
MRI T2 sequence showing intramedullary spinal tumor extending from cervico-medullary junction to C7 level.

**Figure 3:**
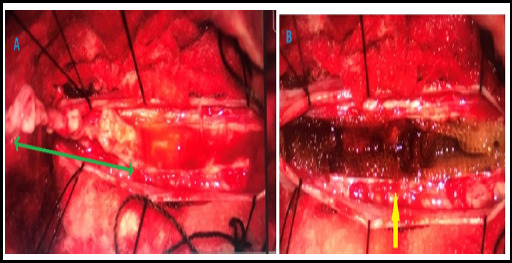
Intraoperative view: Pane A shows the entire length of the tumor (green double arrow) in situ after dissection but before excision, while Pane B depicts the cavity (yellow arrow) left by the tumor following its excision.

This case involves a 32-year-old right-handed male from a rural background who presented to the Neurosurgical OPD with the chief complaint of spastic quadriparesis for 2 months, with preserved bladder and bowel sensations. He had no history of trauma or back pain. Examination revealed motor power of 3/5 in the right upper limb, 0/5 in the left upper limb, and 3/5 in both the right and left lower limbs. Muscle atrophy was evident. Pain, temperature, and vibratory sensations were preserved. The rest of his CNS examinations were within the normal range. An MRI of the spine showed an intramedullary mass extending from the cervico-medullary junction to C7 (Figure 2). The length of the mass was 10 cm. Laminectomy and excision of the intramedullary mass with laminoplasty were performed (Figure 3). Histopathology confirmed it to be an Ependymoma WHO grade 1, and the postoperative McCormick's grade was III.

### Case 3

**Figure 4:**
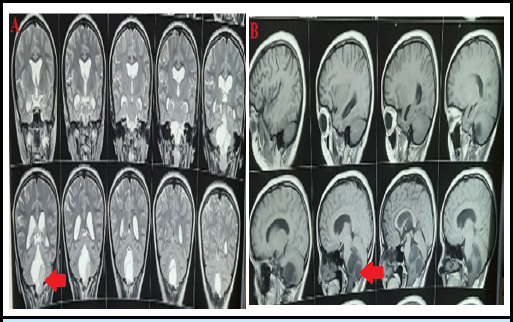
MRI showing posterior fossa tumor extending to the spinal cord in both T2 coronal view (pane A) and T1 sagittal view (pane B).

A 14-year-old right-handed female from a rural area presented with her parents to the Neurosurgical OPD with complaints of imbalance while walking and dizziness for 15 days. She had a history of Ganglioglioma that was operated on 2 years prior at our center. Examination revealed normal motor and sensory functions, but she displayed ataxia while walking. An MRI of the brain with the cervical spine showed a posterior fossa residual mass extending up to the C5 level (Figure 4). Surgical management was planned for the patient, and the postoperative McCormick's grade was I.

## DISCUSSION

**Figure 5:**
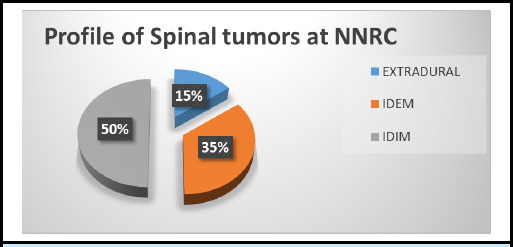
Pie chart depicting the distribution of spinal tumors at NNRC.

Spinal cord tumors are categorized into primary or secondary based on their origin, and further differentiated into extradural and intradural (further classified as extramedullary and intramedullary), according to their location. The majority of intramedullary primary spinal cord tumors are gliomas. Ependymomas, a subtype of glial cell tumors, are the most frequently encountered intramedullary spinal cord tumors, comprising approximately 65% of cases, while astrocytomas account for 30-35%.'^1,4^ We would like to present data procured from the National Neurosurgical Referral Center (NNRC) of Nepal spanning four years from 2019 to 2022 revealed a distinct distribution of spinal tumors. Among the 786 CNS tumors recorded during this period, 38 cases were attributed to spinal tumors. Remarkably, spinal tumors exhibited a distribution pattern distinct from the conventional prevalence observed for such neoplasms, with intradural intramedullary tumors comprising 50% of cases, intradural extramedullary tumors accounting for 35%, and extradural tumors representing 15% of the total (Figure 5). Although the sample size is relatively modest, this distribution could reflect the unique patient population served by the NNRC or possibly emerging trends in the epidemiology of spinal tumors.

The most common presentation among the LIMSCT patient is pain followed by other sensory dysesthesia and motor weakness. Eventually, the bowel and bladder are involved.^1^ Early recognition of symptoms and prompt radiological evaluation resulting in early surgical intervention have shown better outcomes. Thus, maintaining a high index of suspicion is imperative in clinical practice.^1,3^

Contrast-enhanced magnetic resonance imaging (MRI) is the standard choice for evaluation because of its ability to provide clear demarcation of the spinal cord and surrounding structures. Further, subtypes can be identified with greater accuracy which further helps in the planning of surgery^5^. Our Case 1, was found to have a 20.5 cm long intramedullary tumor, which is one of the longest recorded cases of its kind at the NNRC. The presence of such long-level intramedullary tumors presents a distinctive surgical challenge due to their extensive involvement in the spinal cord. Cases of a similar nature have been documented in the literature, including a report by Zhang et al. describing a 300 mm long intramedullary ependymoma extending from the medulla oblongata to the T4 level.^6^

Surgical intervention holds promise for enhanced prognosis and improved neurological function despite the inherent risks of postoperative neurological complications. Therefore, aggressive and complete resection should be the goal whenever possible.3, 6 However, the paramount concern in managing these cases is the preservation of neurological function, which necessitates meticulous surgical planning and execution. Tumor subtype is an important factor that should be considered preoperatively because ependymomas typically afford a favorable surgical plane, facilitating aggressive resection, while astrocytomas, due to their invasive nature, present challenges in achieving complete excision.^1-3^ We performed gross total resection in all three patients as none of them had astrocytoma. Considerable advancements have been achieved in the realm of spinal surgery, notably in microsurgical techniques. Additionally, intraoperative monitoring plays a pivotal role by providing real-time feedback to guide surgical decision-making and minimize the risk of neurological deficits during the procedure.^7,8^ Of particular significance is Case 1 with a 20.5 cm long-level ependymoma, a noteworthy anomaly that likely ranks among the longest intramedullary masses documented in NNRC. This case underscores the remarkable progress in the field of neurosurgery, which continues to refine the management of exceedingly complex spinal tumors. Such advancements offer the potential for more favorable outcomes and improved quality of life for individuals grappling with these challenging conditions.
